# Management of Gunshot Injuries of Mandible with Open Reduction and Internal Fixation versus Closed Reduction and Maxillo-mandibular Fixation

**DOI:** 10.7759/cureus.7830

**Published:** 2020-04-25

**Authors:** Muhammad Muddassar, Rizwan Arshad, Sarah Rabbani, Imran S Qureshi, Imran K Khattak, Zahoor Rana

**Affiliations:** 1 Oral Medicine / Oral and Maxillofacial Surgery, Islam Dental College, Sialkot, PAK; 2 Oral and Maxillofacial Surgery, Bahria University Medical and Dental College, Karachi, PAK; 3 Dentistry, PNS Shifa Hospital, Karachi, PAK; 4 Oral Pathology, Sharif Medical and Dental College, Lahore, PAK; 5 Operative Dentistry, Frontier Medical and Dental College, Abbottabad, PAK; 6 Community Dentistry, Frontier Medical and Dental College, Abbottabad, PAK; 7 Oral and Maxillofacial Surgery, Shaheed Zulfiqar Ali Bhutto Medical University/Pakistan Institute of Medical Sciences (PIMS), Islamabad, PAK

**Keywords:** gunshot injuries, facial fractures, mandibular fractures, closed reduction, open reduction, internal fixation, nonunion

## Abstract

Background/objectives

Gunshot injuries are known to cause severe morbidity and mortality when facial regions are involved. Management of the gunshot wounds of the face comprises of securing an airway, controlling hemorrhage, identifying other injuries and definite repair of the traumatic facial deformities. The objective of the present study was to compare the clinical outcome (infection and nonunion) of open reduction and internal fixation versus closed reduction and maxillo-mandibular fixation (CR-MMF) in the treatment of gunshot injuries of the mandible.

Materials & methods

This study was conducted at Oral and Maxillofacial Surgery Department of Shaheed Zulfiqar Ali Bhutto Medical University/Pakistan Institute of Medical Sciences Islamabad, Pakistan. Ninety gunshot mandibular fractures were randomly allocated in two equal groups. In group-A, 45 patients were treated by open reduction and internal fixation while in group-B, 45 patients were also managed by closed reduction and maxillo-mandibular fixation. Post-operative complications (infection, non-union) were evaluated clinically and radiographically in both groups.

Results

Patients treated by open reduction and internal fixation were having more complications in terms of infection (17.8%) as compared to closed reduction (4.4%) with a p-value 0.044. Whereas non-union was more in closed reduction (15.6%) as compared to open reduction and internal fixation group (2.2%) with a significant p-value 0.026.

Conclusion

Both the treatment modalities can be used in the management of gunshot injuries of mandible and there is need for further studies to have clear guideline in this regard in best interest of patients, community and health care providers.

## Introduction

Injuries to the maxillofacial region present one of the most challenging problems for healthcare professionals worldwide [1]. Trauma to the facial region is commonly caused by road traffic accidents, assaults, gunshots, falls, sports injury and blast injury [2]. Firearm injury contributes to 10% of all maxillofacial trauma and they have exceptionally increased in the recent years due to terrorism and interpersonal violence [3].

Firearms were the third-leading cause of injury-related deaths in United States of America in 2010, following poisoning and road traffic accidents [4]. Firearm injuries are common in Pakistan as well because of instability in the region, easy availability and supply of all kinds of weapons from the tribal areas. Violent crimes, suicidal attempts, accidental release of bullets, domestic violence and air shooting are the main causes of gunshot injuries in Pakistan [5].

Mandibular fractures are one of the most common fractures of the facial skeleton. The most common bony injuries in order of occurrence, associated with gunshot wounds to the face includes the mandible, maxilla and zygomatic bone [6].

Facial gunshot wound patients must be initially managed in accordance with advanced trauma life support (ATLS) protocol [7]. Management plan for a gunshot injury to the face is best devised after characterization of the wound as low or high energy. The surgeon dealing a gunshot injury should consider the concept given by Manson for evaluation of four components - soft tissue injury, bone injury, soft tissues loss, and bone loss. After evaluation of the wound, a decision is made regarding early definitive repair versus the need for delayed repair [8].

Surgical managements of gunshot wounds are divided into three steps: (1) Debridement, (2) fracture stabilization and primary closure, (3) reconstruction of hard tissues with sufficient soft tissue coverage and rehabilitation of the oral vestibule, alveolar ridge and secondary recorrection of deformities [9]. Comminuted mandibular fractures due to gunshot injuries have been treated with various methods which include close or open reduction with semi-rigid like wires osteosynthesis or rigid fixation like plate with screws including external or internal pin fixation [10].

For centuries, closed reduction has been used for the treatment of gunshot mandibular fracture due to their freely availability, less expensiveness of wires and shorter operation time [10]. Historically, it was believed that one should not open firearm wounds because of the risk of compromising blood supply to the bony segments resulting in infection and sequestration. However, this theory was challenged by Kazanjian during the First World War. He stated that “majority of the non-united fractures are due to inadequate immobilization of comminuted fragments of bone, and subsequent infection rather than to initial loss of bone” [10]. Traditionally firearm wounds were managed with conservative debridement, serial dressing changes, external fixation and delayed reconstruction [8].

Nowadays comminuted mandibular fractures due to gunshot have been treated with open reduction and internal fixation using miniplates and reconstruction plates. Open reduction and internal fixation of comminuted mandibular fractures go against the most basic of maxillofacial surgery dogma that states comminuted fractures should be treated closed to prevent stripping the blood supply from the fragments [11]. Past experience with open reduction and internal fixation was not encouraging and many cases of infection reported resulting in significant bone loss and associated morbidity [12].

There are no clear guidelines for the management of facial gunshot injuries due to which large number of patients are suffering with infection, non-union, malocclusion, facial asymmetry and sequestration of devitalized bone. This study is formulated to compare the above mentioned two techniques to correlate which of them has better clinical result and fewer complications, consequently contributing towards the greater goals of a good treatment option and in due process benefit to the concerned patient.

## Materials and methods

This study was carried out in the Department of Oral and Maxillofacial Surgery, Pakistan Institute of Medical Sciences (PIMS) Islamabad, Pakistan. The ethical approval was granted by the Advanced Studies and Research Board (No.F.2-11/SZABMU/AS&RB/47-281), PIMS. Using the WHO sample size calculator, 90 patients were included in the study having gunshot mandibular fracture and divided into two groups: 45 for each treatment modality.

Inclusion criteria

i. Patients of both gender

ii. Age > 15 years

iii. Patients having mandible fracture due to single gunshot injury reported within seven days

Exclusion criteria

i. Cases which require bone grafting

ii. Associated facial fractures other than mandible (excluding condylar fractures)

iii. Edentulous patients

iv. Medically compromised patients

v. Current pregnancy

Data collection procedure

Patients fulfilling the inclusion criteria were selected from the Department of Oral and Maxillofacial Surgery, PIMS. The study protocol and risk-benefit ratio were explained to patients and a written informed consent was taken from each patient. Demographic data such as age, gender and contact details were recorded on a specially designed proforma. The diagnosis was established after taking history, clinical and radiological examination. Standard radiographs, orthopantomogram (OPG), posteroanterior (PA) view face and lab investigation were done in every patient and CT scan with 3D reconstruction (Axial view and coronal view) was also taken preoperatively.

Patients were selected randomly using the table of random numbers generated by Microsoft Excel (Microsoft, Inc. , Redmond, WA, USA) and divided into two groups, “A” and “B”.

i. Group (A) consisting of 45 patients, treated with open reduction and internal fixation (ORIF) using 2.7-mm reconstruction plates through extraoral or intraoral approach under general anesthesia.

ii. Group (B) also comprised of 45 patients, treated under local anesthesia by closed reduction and maxillo-mandibular fixation (Ivy eyelet wiring).

Tooth in the line of fracture was retained only if it was associated with large bony chunk and assists in reduction of fracture and removed if it was non-vital, with root fracture, loose or interfere with fracture reduction. Temporary intraoperative intermaxillary fixation was established in group A. Procedure was done by single surgical team. Maxillomandibular fixation (MMF) was released after reduction and fixation of the fracture with plates and screws. All patients were prescribed injection amoxicillin 500 mg 8-hourly but in case of allergy to amoxicillin injection clindamycin 600 mg 8-hourly, infusion metronidazole 500 mg/100 ml 8-hourly along with intramuscular analgesic diclofenac sodium 75 mg 8-hourly and injection decadron 8 mg 8-hourly were prescribed.

For group B, Ivy loops made with 26 or 24 gauge (≈ diameter 0.4 or 0.5 mm) pre-stretched stainless steel wires were passed between two adjacent stable teeth. Ivy loops were passed between the maxillary teeth and between the mandibular teeth. The MMF was established by passing a 26-gauge straight wire between the loops of upper and lower arch. Immobilization was carried out for six weeks in group B patients. All patients were prescribed syrup amoxicillin 500 mg 8-hourly but in case of allergy to amoxicillin syrup, they were prescribed erythromycin 500 mg 8-hourly, syrup metronidazole 500 mg/100 ml 8-hourly along with analgesic syrup ibuprofen 8-hourly, and were discharged when stable.

Post-operatively infection and non-union were checked in both the groups. Patients were declared infected when there was purulent discharge from the incision or through the sinus tract to the skin or a close swelling that requires an incision and drainage of the purulent material. Infection was checked according to follow up proforma. The fracture site was labeled non-union, when there was abnormal mobility or pain at the fracture site on clinical examination and radiographic (OPG and PA view face) evidence of fracture line as according to Mathog et al. [13].

Osseous union of the fracture was checked clinically and radiographically (OPG and PA view face) at 6th, 12th week and 6th month in both the groups. Patients were advised soft diet in group A and liquid diet in group B. Strict oral hygiene instructions were also given. The variables like age and gender, cause of gunshot injury and location of fractures were also noted in the study.

Statistical analysis

Data were entered and analyzed using SPSS version 20.0 (IBM Corp., Armonk, NY). The continuous numerical variable like age was calculated as means and standard deviations. The categorical variables like gender and complication rate were presented as frequency and percentage in both groups. Complication rates were compared using Chi-square test. A P-value of ≤0.05 was considered significant.

## Results

**Figure 1:**
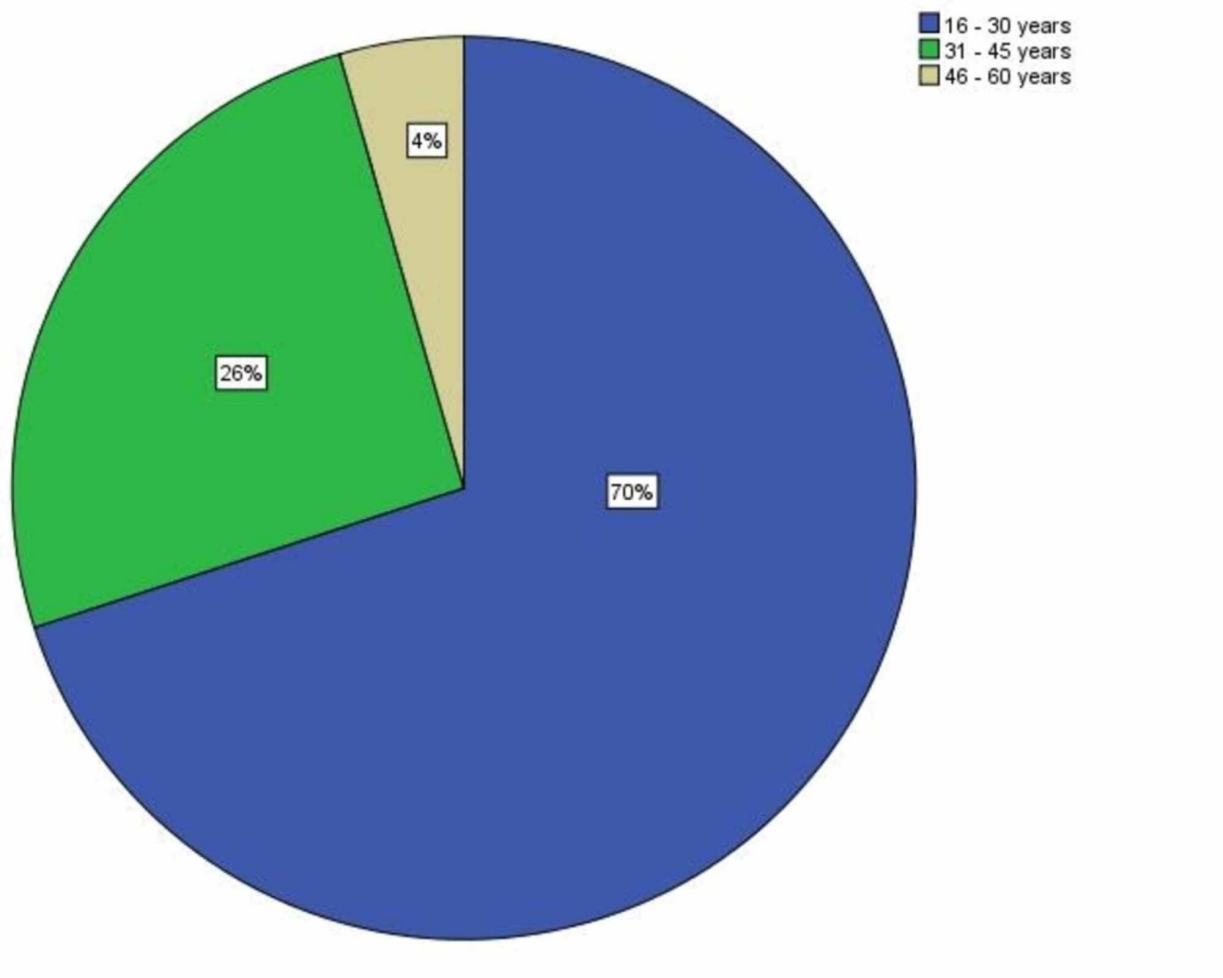
Frequency distribution in terms of age groups of study subjects

Overall, the mean age of the patients was 28.53 ± 8.77 with an age range of 15-55 years. The mean age and standard deviations were also calculated in both the groups as well (Table 1). Age stratification was calculated in terms of frequency and percentages. Majority of the patients (63, 70%) had age range between 16 and 30 years. Similarly, 23 (25.6%) patients had age range between 31 and 45 years. There were four (4.4%) patients who had age range between 46 and 60 years (Figure 1).

**Table 1:** Descriptive statistics of age (years) of patients in both the groups ORIF: Open reduction and internal fixation; CR-MMF: Closed reduction and maxillomandibular fixation.

	Two Groups	N	Mean	Standard Deviation
Age (years)	ORIF (Group A)	45	26.4	6.67
	CR-MMF (Group B)	45	30.67	10.1

Distribution of gender was calculated in terms of frequency and percentages of male and female patients. Total 79 (87.8%) male patients were included in our study. There were 39 (86.7%) males in group A and 40 (88.9%) male patients in group B (Table 2).

**Table 2:** Distribution of gender of patients in both the groups ORIF: Open reduction and internal fixation; CR-MMF: Closed reduction and maxillomandibular fixation.

		Two Groups		Total
		ORIF (Group A)	CR-MMF (Group B)	
Gender	Male	39 (86.7)	40 (88.9)	79 (87.8)
	Female	06 (13.3)	05 (11.1)	11 (12.2)
Total		45	45	90

**Figure 2:**
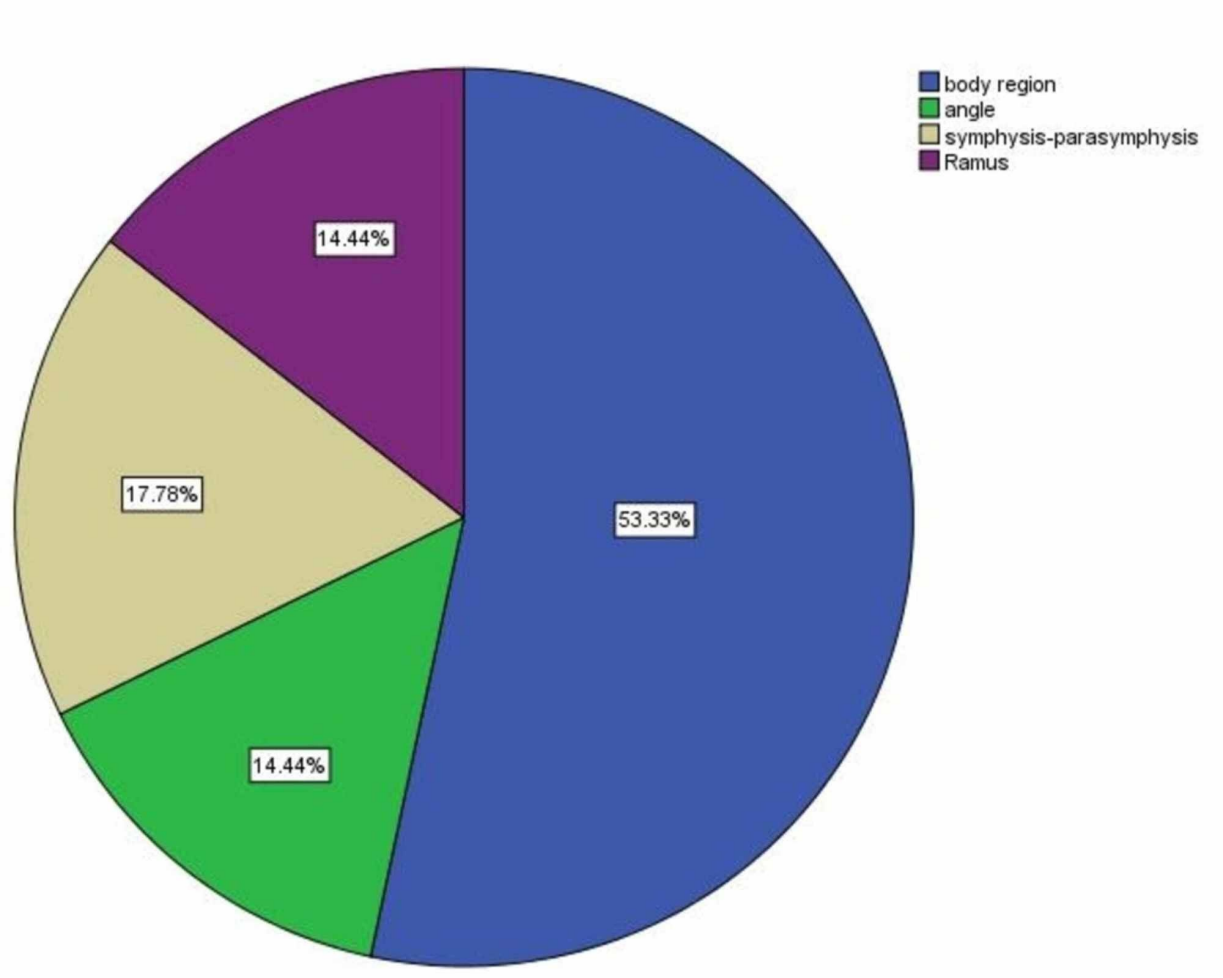
Overall fracture site distribution among study subjects

Comparison of site of fracture was calculated in both the groups. Most of the patients (48, 53.3%) had site of fracture in mandibular body region (Figure 2). Similarly, there were seven (15.6%) patients who had symphysis-parasymphysis site of fracture in group A and nine (20%) patients who had symphysis-parasymphysis fractures in group B (Table 3).

**Table 3:** Frequency of fractured mandibular site in both groups ORIF: Open reduction and internal fixation; CR-MMF: Closed reduction and maxillomandibular fixation.

		Two Groups		Total
		ORIF (Group A)	CR-MMF (Group B)	
Site of fracture	Body region	22 (48.9)	26 (57.8)	48 (53.3)
	Angle	04 (8.9)	09 (20.0)	13 (14.4)
	Symphysis-parasymphysis	09 (20.0)	07 (15.6)	16 (17.8)
	Ramus	10 (22.2)	03 (6.7)	13 (14.4)
Total		45	45	90

Eight patients (17.8%) presented with signs of infection in group A and only two (4.4%) infected patients had been noted in group B. Chi-square test was used to compare infection in both the groups which was statistically significant (p-value 0.044) and showed that complication rate in terms of infection with open reduction and internal fixation was significantly less than with closed reduction and maxillo-mandibular fixation (Table 4).

**Table 4:** Comparison of infection in both the groups ORIF: Open reduction and internal fixation; CR-MMF: Closed reduction and maxillomandibular fixation.

		Two Groups		Total	P-value
		ORIF (Group A)	CR-MMF (Group B)		
Infection	Yes	08 (17.8)	02 (4.4)	10 (11.1)	0.044
	No	37 (82.2)	43 (95.6)	80 (88.9)	
Total		45	45	90	

There were a total of eight patients presented with non-union, one (2.2%) patient in group A and seven (15.6%) patients of non-union in group B. When Chi-square test was used to compare non-union in both the groups it was found to be statistically significant (p-value 0.026) (Table 5).

**Table 5:** Comparison of non-union in both the groups ORIF: Open reduction and internal fixation; CR-MMF: Closed reduction and maxillomandibular fixation.

		Two Groups		Total	P-value
		ORIF (Group A)	CR-MMF (Group B)		
Non-union	Yes	01 (2.2)	07 (15.6)	08 (8.9)	0.026
	No	44 (97.8)	38 (84.4)	82 (91.1)	
Total		45	45	90	

## Discussion

Most of the studies about the treatment of gunshot injuries remain controversial and have not been designed adequately to provide satisfactory comparison of different treatment modalities being used in such patients. Despite this fact, there is relatively little literature available discussing treatment and outcome of these injuries. Controversies continued till today regarding the management of gunshot injuries of mandible among the oral and maxillofacial surgeons.

Considering the age-related findings of the current study, the mean age of the patients involved in this study was 28.53 years (SD± 8.77) with an age range from 15-55 years. Ellis et al. found the mean age to be 30.1 years with an age range of 12-60 years in their study on gunshot comminuted mandibular fractures which is in accordance with this study [10]. Bukhari et al. in their study on management of facial gunshot wound found mean age to be 28 years (SD± 4.98) with an age range of 15-42 years [14]. Channar et al. reported that mean age of patients sustaining gunshot mandibular fractures was 27.36 ± 10.7 years [15]. The higher incidence of firearm injuries in young age group may be explained by the fact that this is the most active phase of life in which the individuals step into the practical life to support their families, facing the challenges and issues of the real world thus being more vulnerable to injuries inflicted by firearms.

Males formed an overwhelming majority of the study population with 87.8% males and 12.2% females. In this study male gender predominated over female. This was consistent with a study conducted by Motamedi [9]. Ellis et al. in their study recorded that 85% (n = 167) of their patients were males and 14% (n = 29) were females similar to our findings [10]. Channar et al. conducted their study on assessment of gunshot maxillofacial injuries and noticed that males were the predominant gender in their study with a number of 94 (78.3%) and female with a number of 26 (21.7%) [16].

Some of the reasons for male predilection are that ours being a male dominated society, male members are more exposed to stress and strains of daily life and are generally more aggressive in demonstrating resistance to perceived threats as compared to females. The fact that males bear the main workload in our society necessitates them to work and travel around quite a lot more than the females [17]. Females appear to be spared due to their household abodes, loyalty to husbands and families and because they hold an honored place even in disputes and enmities. It was also experienced that victims of gunshot injuries were mostly uneducated and belonged to lower socio-economic class [18]. Therefore, it comes as no surprise that this study population is also male dominated.

Most common site of fracture noted in this study was mandibular body region with 53.3% followed by symphysis-parasymphysis (17.8%), angle (14.4%) and ramus region (14.4%). Newlands et al. reported the mandibular body (38.8%) as the most common fracture site in the mandible followed by angle and then the anterior region [12]. Similar findings have been reflected in other studies as well [10]. The reason for common fracture in the body area could be due to large surface area of the mandibular body.

Post-operative complications, infection and non-union were evaluated in this study. The main enigma faced by researchers while assessing the literature is that the tools to measure infection and to gauge its prevalence is hard to compare from one study to the other. Malanchuk and Kopchak define infection as an inflammatory disease, caused by microorganisms after mandibular fractures; infection consists of a group of suppurative inflammatory diseases that are caused by oral flora post-traumatically. The main types of infections are post-traumatic osteomyelitis (either acute or chronic), paramandibular abscess, phlegmons, infected hematomas, and infected wounds [19].

In the present study there were 10 patients who developed infection during complete follow-up. Out of these 10 patients, eight (17.8%) belonged to ORIF group and two (4.4%) to CR-MMF group. Chi-square test was used to compare infection in both the groups which was statistically significant (p-value 0.044). Postoperative infection is perhaps difficult to determine whether it arises from the injury itself or from the treatment. We have experienced more cases of infection in ORIF (08) as compared to CR-MMF (02). ORIF needs incision, flap elevation and periosteal stripping in comminuted mandibular fractures. This in turn further decreases vascularity owing to periosteal elevation and increases the possibility of wound dehiscence and contamination. Extensive periosteal stripping may decrease the resistance to infection.

Infection is also related to the degree of severity of fracture. Gunshot injuries are often associated with increased bone fragmentation and soft tissue disruption, which could be easily linked to wound contamination and subsequent infection. We have observed that infection was found in more comminuted fracture and it has been reflected in other gunshot studies as well [10,12]. The results of present study regarding postoperative infection are comparable with other local and International data. Neupert and Boyd conducted a study on a series of 32 low velocity gunshot wounds to the mandible. They reported 27% infection rate by ORIF which is high as compared to our study. They mentioned in their study that the reason for higher rate of infection was initial extensive hard and soft tissue injuries which is similar to our findings [20]. Channar et al. carried out prospective study to determine the outcome of ORIF and CR-MMF. They included those gunshot patients in their study who had continuity defect less than 1 cm. They reported infection rate in ORIF and CR-MMF as 16.6% and 10%, respectively. They concluded that ORIF is a better treatment option as compared to CR-MMF but careful selection of the patient is very important. Our results regarding infection rate in ORIF and MMF are almost in accordance with those of Channar et al. [15].

The next complication recorded in our study after infection was non-union. It means that when bone healing does not occur at a fracture site. There is currently no accepted standardized definition of non-union in literature, especially the time frame of a non-union is not clearly defined. However, we considered non-union as any fracture which fails to unite within three month (confirmed radiographically with OPG and PA View) and shows abnormal movement or pain at the site of fracture. Non-union has been reported as 0%-5% of mandibular fractures in literature in dentate patients, whereas incidence in edentulous patients is 10%-15% [13].

Non-union was present in eight cases. There was one (2.2%) case of non-union in ORIF group and seven (15.6%) cases in CR-MMF group. Chi-square test was used to compare non-union in both the groups which was statistically significant (p-value 0.026).

In our study, three cases of infection later developed non-union, one case from ORIF group and two cases from CR-MMF. Infection results in a hypoxic environment, which may lead to fibrous union without bone formation. A high association between infection and non-union has been reported. Mathog et al. reported that 17 out of 25 cases of non-union were associated with infection [13]. Malanchuk and Kopchak augmented this finding in a study of 195 infected mandible cases and showed that 55% of those developed non-union secondary to infection [19].

Ellis et al. performed 10-year study on 198 comminuted mandible fractures. Thirty-six of those were gunshot injury cases and were mainly treated by MMF or external pin fixation. He had documented five cases with non-union after closed method which is almost similar to our findings. They discouraged the use of CR-MMF in such cases [10]. Channar et al. in 2011 reported non-union rate in ORIF and CR-MMF respectively as 3.3% and 20% which also favours our study [15].

As in other studies, present study has also some limitations to mention. One of the limitations of this study was probably the smaller number of patients with only two variables. Studies conducted over large number of patients and more number of variables would have allowed the reader to have a more conclusive results. Age of the patient was recorded in number of years as told by patient himself, instead of calculating from patient’s date of birth; it may lead to discrepancies in data collection and misleading results regarding mean age and the age range. Habits of patient (smoking and drinking) may affect soft tissue and bony healing, predisposing the fracture site to development of infection.

As with any debate in the practice of surgery, there must be no absolutes. Each patient deserves the attention of directed thought and treatment which his or her individual problem demands. Adhering blindly to algorithms and approaching treatment options closed-mindedly produces only average results most of the time and poor results too frequently. Creative thinking based on sound surgical principles coupled with good clinical judgment drives excellent patient care outcomes and advancement of new techniques.

## Conclusions

The findings of the current study demonstrated that the frequency of post-op infection rate is lower in conservative, closed reduction and fixation. On the other side, rigid internal fixation offers many advantages to the patient and is claimed to be superior to conventional techniques in spite of slightly higher infection rates. Both the treatment modalities can be used in the management of gunshot injuries of the mandible and there is a need for further studies to have clear guidelines in this regard in the best interest of patients, community and health care providers.

## References

[1] Schaftenaar E, Bastiaens GJ, Simon EN, Merkx MA (2009). Presentation and management of maxillofacial trauma in Dar es Salaam, Tanzania. East Afr Med J.

[2] Khan SU, Khan M, Khan AA, Murtaza B, Maqsood A, Ibrahim W, Ahmed W (2007). Etiology and pattern of maxillofacial injuries in the Armed Forces of Pakistan. J Coll Physcian Surg Pak.

[3] Cheema SA, Ameen F (2006). Incidence and causes of maxillofacial skeletal injuries at the Mayo Hospital in Lahore, Pakistan. Br J Oral Maxillofac Surg.

[4] (2020). National Center for Injury Prevention and Control, U.S. Centers for Disease Control and Prevention. https://www.cdc.gov/about/leadership/leaders/ncipc.html.

[5] Hussain Z, Mujahid MM, Afridi HK, Arif M (2006). Homicidal deaths by firearms in Peshawar: an autopsy study. J Ayub Med Coll Abbottabad.

[6] Jett HH, Von Hoy JM, Hamit HF (1972). Clinical and socioeconomic aspects of 254 admissions for stab and gunshot wounds. J Trauma.

[7] Kaufman Y, Cole P, Hollier LH Jr (2009). Facial gunshot wounds: trends in management. Craniomaxillofac Trauma Reconstr.

[8] Thorne CH (1992). Gunshot wounds to the face. Current concepts. Clin Plast Surg.

[9] Motamedi MHK (2003). Primary management of maxillofacial hard and soft tissue gunshot and shrapnel injuries. J Oral Maxillofac Surg.

[10] Ellis E III, Muniz O, Anand K (2003). Treatment considerations for comminuted mandibular fractures. J Oral Maxillofac Surg.

[11] Baurmash HD (2004). Closed reduction, an effective alternative for comminuted mandible fractures. J Oral Maxillofac Surg.

[12] Newlands SD, Samudrala S, Katzenmeyer WK (2003). Surgical treatment of gunshot injuries to the mandible. Otolaryngol Head Neck Surg.

[13] Mathog RH, Toma V, Clayman L, Wolf S (2000). Nonunion of mandible: an analysis of contributing factors. J Oral Maxillofac Surg.

[14] Sali Bukhari SG, Khan I, Pasha B, Ahmad W (2010). Management of facial gunshot wounds. J Coll Physicians Surg Pak.

[15] Channar KA, Dal AQ, Safia Safia, Warriach RA (2011). Comparison of open reduction and internal fixation versus closed reduction and maxillomandibular fixation for the treatment of gunshot injuries of mandible. J Liaquat Uni Med Health Sci.

[16] Channar KA, Riaz N, Alam J, Warriach RA, Memon AB (2012). An assessment of maxillofacial gunshot injuries and emergency management. J Pak Dent Assoc.

[17] Shah MM, Ali U, Fasseh-uz-Zaman Fasseh-uz-Zaman (2008). Morbidity & mortality of firearm injury in Peshawar region. J Ayub Med Coll Abbottabad.

[18] Hussain T, Tajammul N, Bhatti MA, Hanif S (2005). Firearm injuries - a study of 110 cases. Ann King Edward Med Coll.

[19] Malanchuk VO, Kopchak AV (2007). Risk factors for development of infection in patients with mandibular fractures located in the tooth-bearing area. J Craniomaxillofac Surg.

[20] Neupert EA, Boyd SB (1991). Retrospective analysis of low-velocity gunshot wounds to the mandible. Oral Surg Oral Med Oral Pathol.

